# Influence of Rare Earth Element (Y) on Microstructure and Corrosion Behavior of Hot Extrusion AZ91 Magnesium Alloy

**DOI:** 10.3390/ma13163651

**Published:** 2020-08-18

**Authors:** Yanan Cui, Yonghai Wang, Zhongyu Cui, Wenlong Qi, Jidong Wang, Pengfei Ju, Yang Zhao, Bin Liu, Tao Zhang, Fuhui Wang

**Affiliations:** 1 Corrosion and Protection Laboratory, Key Laboratory of Superlight Materials and Surface Technology, Harbin Engineering University, Ministry of Education, Harbin 150001, China; yncui1992@163.com (Y.C.); fhwang@mail.neu.edu.cn (F.W.); 2 Technical Office Department, Beijing Institute of Space Long March Vehicle, Beijing 100076, China; jingying_519@sina.com; 3 School of Materials Science and Engineering, Ocean University of China, Qingdao 266100, China; cuizhongyu@ouc.edu.cn; 4 Shenyang National Laboratory for Materials Science, Northeastern University, Shenyang 110819, China; 1910215@stu.neu.edu.cn (W.Q.); wangjidong@stumail.neu.edu.cn (J.W.); zhaoyang7402@mail.neu.edu.cn (Y.Z.); 5 Shanghai Aerospace Equipment Manufacture, Shanghai 200245, China; jupengfei10@163.com

**Keywords:** AZ91 Mg alloy, rare earth element, microstructure, corrosion resistance

## Abstract

The influence of rare earth element (RE) Y on the microstructure and corrosion behavior of extruded AZ91 Mg alloy was surveyed via morphology characterization and corrosion performance measurements. The results indicate the corrosion resistance of the transversal section of AZ91 Mg alloy containing Y was improved compared with AZ91 Mg alloy without Y. The corrosion resistance of the longitudinal section of AZ91 Mg alloy with Y was lower than that of AZ91 Mg alloy without Y. The change of corrosion resistance can be attributed to the dispersion and volume fraction of the second phase, the effect of cathodic reduction rate, and the refined second phase.

## 1. Introduction

Magnesium (Mg) and its alloys, as one of the most potential materials to replace aluminum alloys, are widely applied in all kinds of fields, such as the automotive industry, aerospace components, and electronics, due to their ultra-light weight, high weight-to-strength ratios, great specific strength, remarkable anti-shock resistance, and excellent recyclability in recent years [1,2,3,4,5,6,7,8]. Unfortunately, their intense chemical reaction activity and relatively low corrosion resistance impede their further applications [9,10]. It is essential to optimize the corrosion resistance of Mg and its alloys. This has attracted much attention from many researchers and has become an awfully vital topic in the practical application of Mg alloys.

There are many ways to protect Mg alloys from corrosion [11,12,13,14,15,16,17]. Among these corrosion protection methods, changing the microstructure and composition of Mg alloys by alloying is one of the widely employed manners in the study of corrosion protection of Mg alloys. This method has proven to be effective [18,19,20,21,22]. Rare earth elements (RE) have become the considerable alloying elements of corrosion protection research works of Mg alloys [16,17,23,24,25]. T. Takenaka et al. [23] suggested the corrosion resistance of Mg alloys was improved by adding a small quantity of RE, whereas an excess of RE degenerated the corrosion resistance of Mg alloys. They also found that surface oxide films containing Mg and RE can provide desired corrosion resistance to Mg alloys. Hu et al. [26] surveyed the microstructure and corrosion behavior of Mg-4Al-3Ca alloy after adding mixed Y and Ce. The results indicated that adding the mixture of Y and Ce to Mg-4Al-3Ca alloy refined the thick strip (Mg, Al)_2_Ca phase and changed the continuity of the (Mg, Al)_2_Ca phase, and the newly obtained distributed granular Al-RE phase formed a barrier layer in the matrix. The improved corrosion resistance is due to the combination of a good corrosion barrier, moderate precipitate phases, and pieces of mixed Y and Ce.

Extrusion is an important and effective method to change the mechanical performances of Mg alloy [27,28,29]. However, the grain size and dislocation density have a significantly important effect on the corrosion inhibition property of Mg alloy. Deng et al. [27] studied the influence of extrusion on the corrodibility and microstructure properties of Mg-2Ca-χAl (χ = 0, 2, 3, 5 wt.%) alloys. The results showed the grains were obviously refined and the local corrosion occurred in extruded Mg alloy compared with cast Mg alloy. W. J. Kim et al. [30] researched the influence of indirect extrusion processes on microstructure, mechanical capacities, and corrosion of the pure Mg and Mg-Ca alloys. The results indicated the improved corrosion resistance of extruded Mg-Ca alloys with Ca ≥ 1% was mainly attributed to effective break-up of (semi-)continuous secondary phase (Mg_2_Ca) existing along grain boundaries or interdendritic regions during extrusion. Eliezer et al. [31,32] disclosed that extrusion reduced the corrosion inhibition property of AZ80 and AZ31 Mg alloys. Now, scholars have done a lot of investigations on the influences of RE on the corrosion property of Mg alloys and the influence of extrusion on the mechanical properties of Mg alloys. Yet, there is not enough research on the influence of RE on the corrosion behavior of extruded Mg alloys. In addition, it is not clear the effect of RE on the corrosion resistance of extruded Mg alloys at present. Therefore, it is very essential to survey the impact of RE on the corrosion behavior of extruded Mg alloys on further study.

Our group [17] reported the effect of Nd on the microstructure and corrosion behavior of extruded AZ91 Mg alloy (Ex-AZ91 alloy). The results manifested that the twins and dislocation decreased and the corrosion resistance increased after adding Nd. Nd and Y are rare earth elements. However, the standard electrochemical potential of Y (−2.372 V vs. SHE) is quite unusual, which is equal to that of Mg (−2.372 V relative to SHE). The influence of Y on the microstructure and corrosion resistance attracted our interest. Extruded AZ91 Mg alloy with addition of 1.5 wt.% Y (Ex-AZ91-Y alloy) and extruded AZ91 Mg alloy without the addition of Y (Ex-AZ91-free alloy) were selected as experimental materials in this study. The corrosion resistance and microstructure of Ex-AZ91-Y alloy were investigated. The correlation of microstructure and corrosion behavior was established, and the influence and mechanism of Y on the corrosion resistance of Ex-AZ91 alloy were clarified. Furthermore, a simplified model for the second phase and micro galvanic corrosion density was established.

## 2. Experimental Section

### 2.1. Materials and Solutions

The hot extrusion AZ91 Mg alloy ingot was used in this study. AZ91 Mg alloy was supplied by Tianjin Dongyi Mg Products Co., Ltd. (Tianjin, China). Sodium chloride (NaCl ≥ 99.8%), magnesium hydrate (Mg(OH)_2_, 98%), chromium trioxide (CrO_3_ ≥ 99.8%), and silver nitrate (AgNO_3_ ≥ 99.8%) were purchased from Sinopharm Chemical Reagent Co., Ltd. (Shanghai, China). The hot extrusion Mg alloy with and without the addition of Y was extruded in 250 °C. The horizontal water press was used to squeeze AZ91-free and AZ91-Y alloy with the extrusion rate of 3–5 m/s and the ratio of 18:1. The chemical compositions of Ex-AZ91-free and Ex-AZ91-Y alloy are listed in Table 1.

Weight loss experiments and electrochemical measurements include transversal section coupons and longitudinal section coupons of Ex-AZ91 and Ex-AZ91-Y alloy. For the electrochemical test, coupons were cut into cylinders (φ 12.6 mm × 10 mm). The specimens were embedded in epoxy resin, leaving an exposed surface as the working electrode surface. The back of the working electrode surface was linked to a copper wire to form an electrical pathway. Before electrochemical measurements, the working surfaces of all the specimens were ground using 240, 600, 1000, and 2000 mesh of sandpapers, in turn, rinsed with acetone and deionized water, and dried using a hair dryer.

For weight loss measurements, the size of the transversal section specimens was φ 12.6 mm × 3 mm, and the size of the longitudinal section coupons was 9 mm × 8 mm × 3 mm.

A 3.5 wt.% NaCl solution containing saturated Mg(OH)_2_ was used during the entire process of the experiment. Saturated Mg(OH)_2_ was added to maintain the pH value constant. All experiments were carried out under 25 ± 1 °C; for better reproducibility, the number of all weight loss tests and electrochemical measurements was no fewer than three under 25 ± 1 °C.

### 2.2. Microstructure Examination

The microscopic morphologies of Ex-AZ91-free and Ex-AZ91-Y alloy specimens were detected by scanning electron microscope (SEM, QUANTA 200 F, Philips, Amsterdam, The Netherlands) equipped with energy disperse X-ray spectrometer (EDS) and transmission electron microscope (TEM, TECNAI G2 F3, FEI Co., Hillsboro, OR, USA). The work function of TEM is 300 kV. The corrosion morphologies of Ex-AZ91-free and Ex-AZ91-Y alloy after immersing for 12 h were also observed by scanning electron microscope.

An X-ray diffraction spectrometer (XRD, X’Pert Pro, Malvern Panalytical Ltd., Malvern, UK) with Cu Kα as the excitation source was used to determine the phase of extruded Mg alloy. The working voltage and working current of XRD were 40 kV and 20 mA, respectively, and the scan range was 20–100°.

### 2.3. Electrochemical Measurement

Electrochemical tests were conducted in a three-electrode cell with a platinum foil as the counter electrode, an Ag/AgCl electrode (saturated potassium chloride) as the reference electrode, and the two Ex-AZ91 alloys as the working electrode, using the Zahner Zennium electrochemical workstation (Zahner, Kronach, Germany).

Potentiodynamic polarization measurement was conducted over a potential range from −300 mV vs. open circuit potential until the range of current density reached between 1 and 10 mA with a scan rate of 0.333 mV/s. Electrochemical impedance spectroscopy (EIS) was carried out with a frequency range from 100 kHz to 0.01 Hz using ac signals of amplitude 5 mV peak to peak at open circuit potential; the data were fitted by using the commercial software ZsimpWin (Princeton Applied Research, Oak Ridge, TN, USA).

### 2.4. Weight Loss Measurement

The pretreatment samples were weighed before immersion using analytical balance with a precision of 0.00001 g for the original weight and its size was measured. The samples were immersed in the 3.5 wt.% NaCl solution for 12 h under 25 ± 1 °C. Then, the specimens corroded for 12 h were soaked in a chromate solution (200 g/L CrO_3_ + 10 g/L AgNO_3_) for 10 min. This operation was carried out to remove the corrosion products of the samples. In the end, the specimens were washed with deionized water and ethyl alcohol, dried by hot air flow, and reweighed to obtain the weight loss. The equation of corrosion rate can be calculated as follows [33]:

(1) $$V_{corr}\;=\;\frac{W_{0}\;-\;W_{1}}{At}$$

where *V_corr_* is the corrosion rate of the sample, g·m^−2^·h^−1^; *W*_0_ is weight of the sample before corrosion, g; *W*_1_ is weight of the sample after removing corrosion product, g; *A* is the surface area of the sample, m^2^; *t* is the immersion time, h.

## 3. Results

### 3.1. Scanning Electron Microscopy

**Figure 1:**
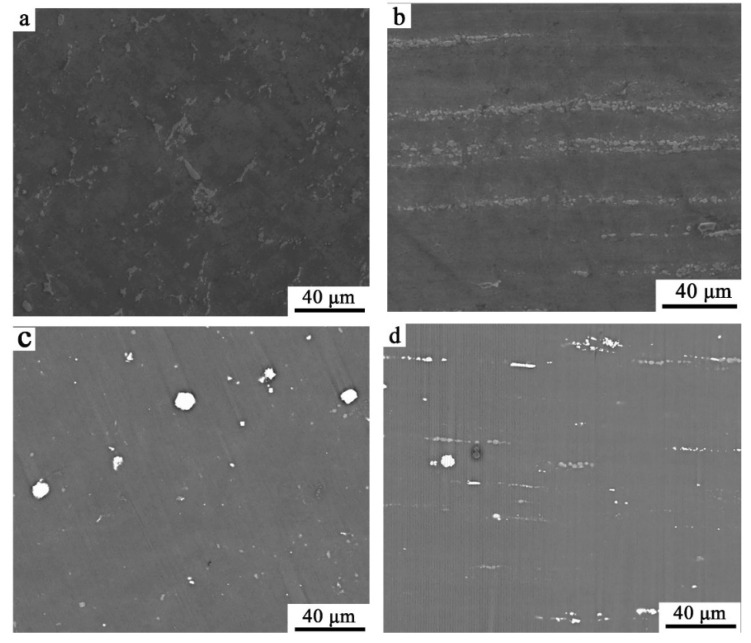
Microstructure morphologies of (**a**) transversal section and (**b**) longitudinal section of Ex-AZ91-free alloy; (**c**) transversal section and (**d**) longitudinal section of Ex-AZ91-Y alloy.

The morphologies of the transversal section and longitudinal section of Ex-AZ91-free and Ex-AZ91-Y alloy are exhibited in Figure 1. The second phase of the transversal section in EX-AZ91 Mg alloy was dispersive distribution (Figure 1a,c); the β phase of the longitudinal section of Ex-AZ91-free and Ex-AZ91-Y alloy dispersed along the extrusion direction with a line (Figure 1b,d), which is consistent with the previous results [17]. The volume fractions of the second phase of Ex-AZ91 alloy with and without the addition of Y are shown in Table 2. The results manifest that after adding Y, the volume fraction of the second phase distinctly decreased.

**Figure 2:**
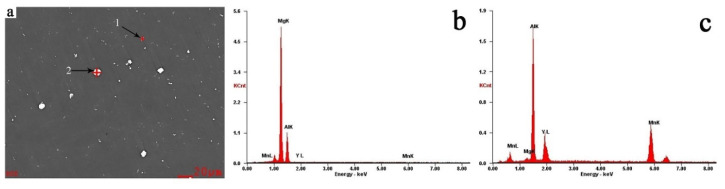
EDS spectrums of Ex-AZ91 alloy. (**a**) SEM image, (**b**) point 1, and (**c**) point 2.

To ensure the compositions of the white granular second phase, the compositions were detected by EDS. The results are exhibited in Figure 2 and Table 3. It can be seen that the darker second phase (point 1) primarily consists of Mg and Al elements, which is the dispersed β phase (Figure 2). The white granular second phase (point 2) mainly contains Al, Mn, and Y elements, which may be the Al-Mn-Y phase. The reduction in the second phase indicates that the new phase was formed between Al, Mn, and Y; a part of Al and Mn element was solidly dissolved into the matrix.

### 3.2. Transmission Electron Microscopy

**Figure 3:**
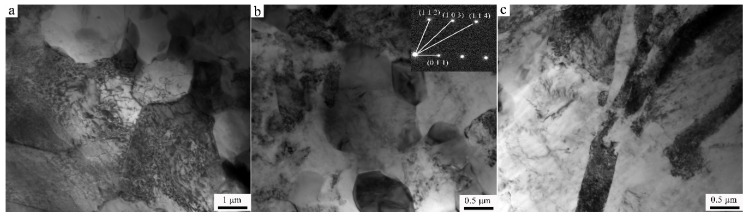
TEM images of Ex-AZ91-free alloy (**a**) refined grain with higher dislocation, (**b**) β phase, and (**c**) twins.

**Figure 4:**
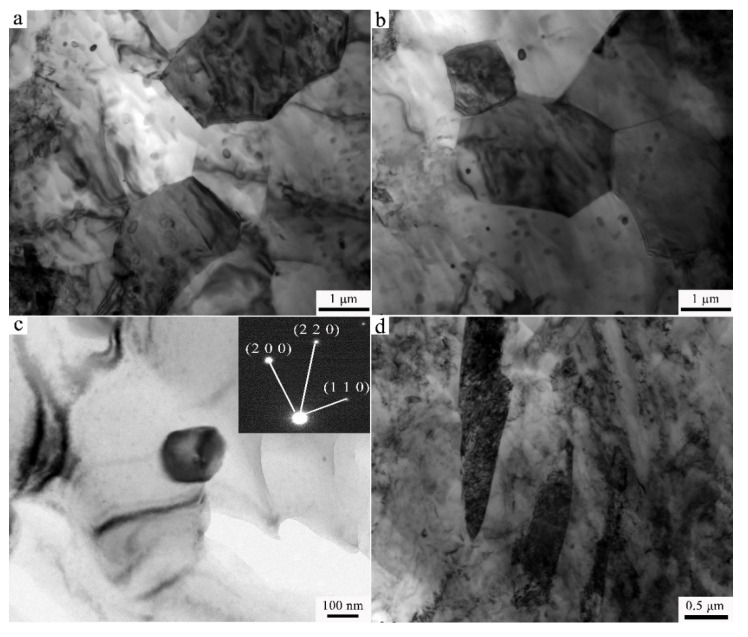
TEM images of Ex-AZ91-Y alloy, (**a**) refined grain, (**b**) recrystallization grain, (**c**) Al-Mn-Y phase, and (**d**) twins.

In order to better understand the effect of Y on the microstructure of Ex-AZ91 alloy, TEM was performed. The results are shown in Figure 3 and Figure 4. As presented in Figure 3a, prior to the addition to Y, there are the sizes of the grains approximately 3–5 μm in Ex-AZ91-free alloy. The dislocation density is relatively high. The β phase is refined and the grain boundary of the α phase that exists precipitates (Figure 3b). There are a lot of twins in Ex-AZ91-free alloy (Figure 3c). However, after adding RE, the sizes of grains did not change significantly and still remained about 3–5 μm (Figure 4a). Fine recrystallized grains and equiaxial crystals appeared and the dislocations were not observed in Ex-AZ91-Y alloy (Figure 4b). Compared with Ex-AZ91-free alloy, the β phase of Ex-AZ91-Y alloy significantly reduced and was replaced by tiny Al-Mn-RE phase (Figure 4c). The size of the Al-Mn-RE phase is about 100 nm. Moreover, after the addition of Y, only a few twins were found (Figure 4d).

### 3.3. X-ray Diffraction Spectrometry

**Figure 5:**
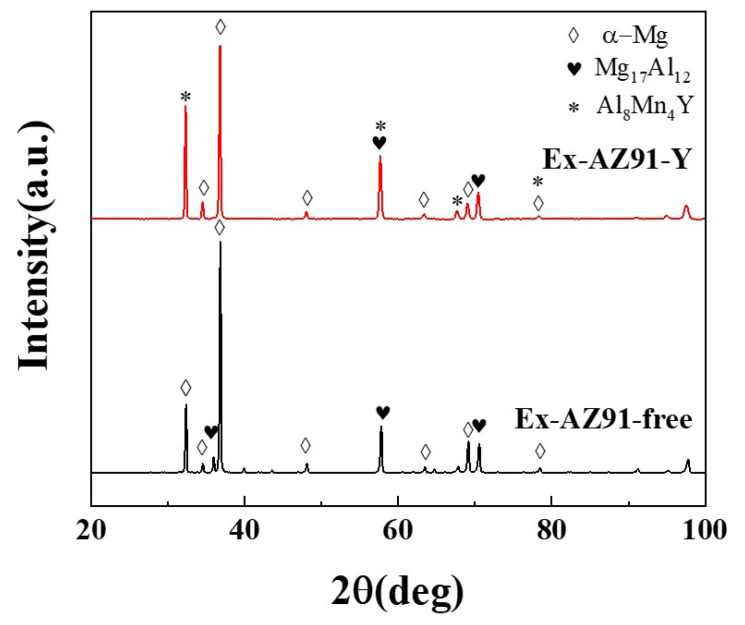
X-ray diffraction spectrum of Ex-AZ91 alloy before and after adding Y.

Figure 5 presents the XRD patterns of Ex-AZ91 alloy with and without Y. The intensity of peaks correspond to α-Mg and Mg_17_Al_12_ in Ex-AZ91-free alloy (Figure 5 black line). The peaks of Ex-AZ91-Y alloy are α-Mg, Mg_17_Al_12_, and Al_8_Mn_4_Y (Figure 5 red line). After the addition of Y, the diffraction peak of the β phase of Mg alloy decreased and a new diffraction peak obviously appeared. The new diffraction peak manifests Y, Al, and Mn elements to form the Al-Mn-RE phase. For Ex-AZ91-Y alloy, the Al_8_Mn_4_Y phase was formed.

### 3.4. Weight Loss Measurement

**Figure 6:**
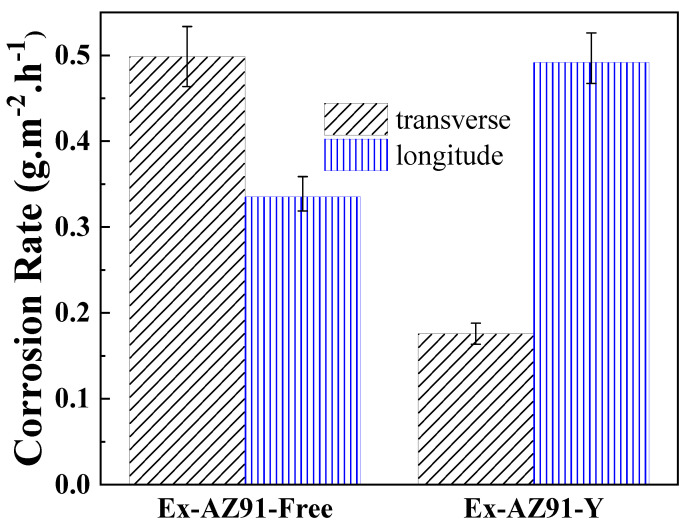
Corrosion rate of the transversal and longitudinal sections of Ex-AZ91-free and Ex-AZ91-Y alloy after immersed 12 h.

Generally speaking, for the determination of corrosion rates, the weight loss test is one of the most accurate and precise methods [34,35]. The weight loss measurements were used to demonstrate the corrosion resistance of two different extruded Mg alloys. The results are exhibited in Figure 6. The corrosion rates of the transversal and longitudinal section of Ex-AZ91-free alloy are 0.4987 and 0.3354 g/m^2^·h. The corrosion rates of the transversal and longitudinal sections are 0.1758 and 0.4918 g/m^2^·h for Ex-AZ91-free alloy. For the transversal section, the corrosion rate of Ex-AZ91-Y alloy reduces significantly. For the longitudinal section of Ex-AZ91-Y alloy, the corrosion rate accelerates distinctly.

### 3.5. Potentiodynamic Polarization Curve

**Figure 7:**
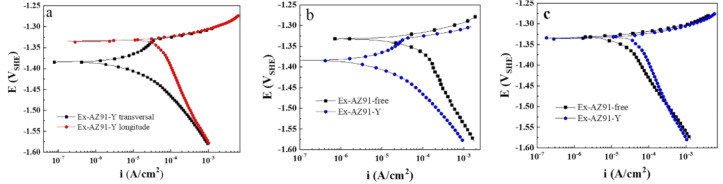
Polarization curves of (**a**) transversal section and longitudinal section of Ex-AZ91-Y alloy, (**b**) transversal section, and (**c**) longitudinal section of Ex-AZ91 alloy before and after the addition of Y.

In order to investigate the corrosion resistance of Ex-AZ91-Y alloy, the polarization curves of the longitudinal and transversal sections of Ex-AZ91 alloy with and without adding Y are illustrated in Figure 7. It can be seen from Figure 7a that the transversal section exhibited weak passivation behavior and the cathodic hydrogen evolution process was inhibited compared with the polarization curve of the longitudinal section in the Ex-AZ91-Y alloy. The corrosion current densities of the transversal section and longitudinal section were 115 and 520 mA/cm^2^, respectively. Therefore, the corrosion current density obtained by Tafel fitting manifested the corrosion resistance of the transversal section better than that of the longitudinal section in Ex-AZ91-Y alloy.

This shows the polarization curves of the transversal section of Ex-AZ91-free alloy revealed active dissolution and the anodic branch of Ex-AZ91-Y alloy appeared a weak passivation region (Figure 7b). Besides, the corrosion current density of the transversal section of Ex-AZ91-Y alloy (115 mA/cm^2^) declined obviously compared with the transversal section of Ex-AZ91-free alloy (632 mA/cm^2^). The results indicate the corrosion resistance of the transversal section of Ex-AZ91-Y alloy is superior to that of Ex-AZ91-free alloy.

Figure 7c depicts that there is not much difference among the anodic branch of the longitudinal section of two extruded Mg alloys, and both of them showed active dissolution. The difference in corrosion resistance can be attributed to the change of cathodic polarization curves. According to the cathodic branch of the polarization curve, the hydrogen evolution process of Ex-AZ91-Y alloy is promoted, which results in the poor corrosion resistance of the longitudinal section of Ex-AZ91-Y alloy. Besides, the corrosion current densities of Ex-AZ91-free and Ex-AZ91-Y alloy are 191 and 520 mA/cm^2^, respectively. The results manifest the corrosion resistance of the longitudinal section of Ex-AZ91-free alloy is superior to that of Ex-AZ91-Y alloy. To further ascertain the influence of Y on the corrosion resistance of Ex-AZ91 alloy, the EIS was carried out.

### 3.6. Electrochemical Impedance Spectroscopy

**Figure 8:**
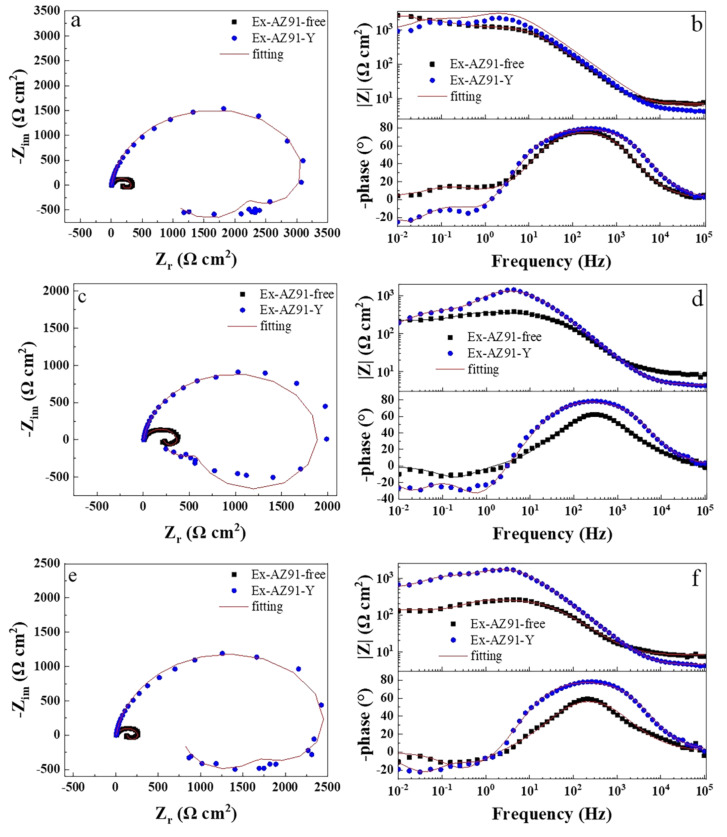
Nyquist and Bode plots of the transversal section of Ex-AZ91-free and Ex-AZ91-Y alloy, (**a**,**b**) 2 h, (**c**,**d**) 8 h, and (**e**,**f**) 12 h.

**Figure 9:**
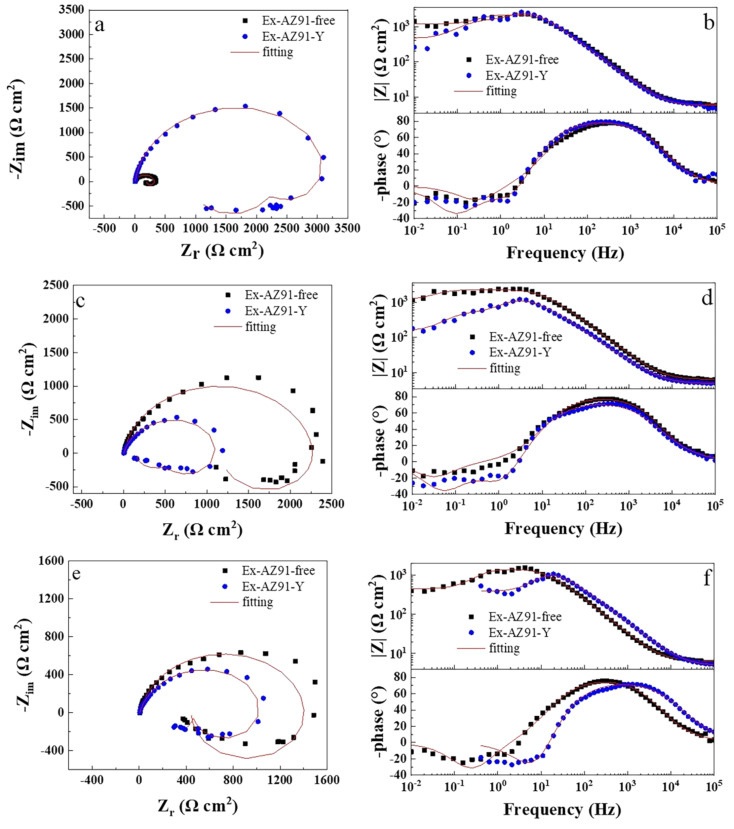
Nyquist and Bode plots of the longitudinal section of Ex-AZ91-free and Ex-AZ91-Y alloy in the different soaking time, (**a**,**b**) 2 h, (**c**,**d**) 8 h, and (**e**,**f**) 12 h.

**Figure 10:**
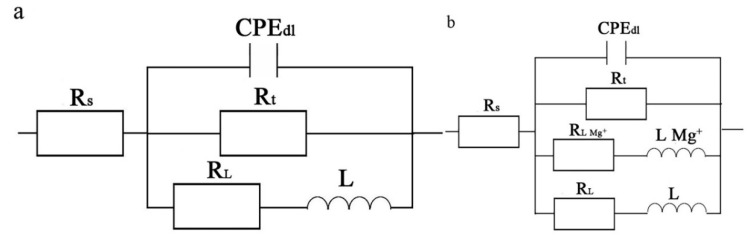
Equivalent circuit of Ex-AZ91-free alloy (**a**), Ex-AZ91-Y alloy (**b**).

Figure 8 and Figure 9 describe the EIS of the transversal and longitudinal sections of Ex-AZ91-free and Ex-AZ91-Y alloy for different immersion times, respectively. Ex-AZ91-free alloy exhibits one induced loop in the low frequency and a capacitive loop in the high frequency, while Ex-AZ91-Y alloy contains two induced loops in middle and low frequencies and a capacitive loop in high frequency. The equivalent circuit models are presented in Figure 10. According to Figure 8, it can be found that the impedance loop of the transversal section of Ex-AZ91-Y alloy is larger than that of Ex-AZ91-free alloy, which demonstrates the corrosion resistance of the transversal section of Ex-AZ91-Y alloy is improved obviously. Figure 9 exhibits that the impedance loop of the longitudinal section of extruded Mg alloy decreased gradually after adding Y in the course of soaking, indicating the reduced corrosion resistance of the longitudinal section in Ex-AZ91-Y alloy.

In an equivalent circuit model (Figure 10), the constant phase element (CPE) replaced the perfect capacitors, which represents nonideal capacitive behavior in the surface of the electrodes. R_s_ is the solution resistance, CPE_dl_ is the double layer capacitance, R_t_ is the charge transfer resistance, R_L_ is the resistance of inductance, and L is the inductance, which is due to the rupture of oxide or hydroxide film. In the equivalent circuit model in Figure 10b, L_Mg+_ is the inductance of the Mg^+^ reaction on the breaking area of partial protective film and R_L, Mg+_ is the resistance of the Mg^+^ reaction [17,36].

**Figure 11:**
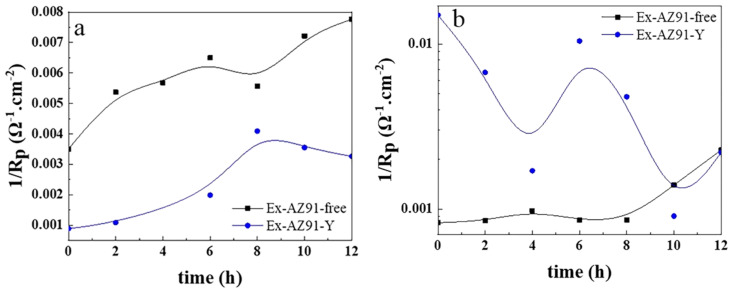
1/Rp of (**a**) transversal and (**b**) longitudinal section of Ex-AZ91-free and Ex-AZ91-Y alloy during various immersion times.

The curve of the reciprocal of polarization resistance (*R*_p_) 1/*R*_p_ with immersion time in Figure 11 is obtained according to the equivalent circuit (Figure 10). The 1/*R*_p_ is proportional to corrosion rate under the corrosion potential. It can be seen from Figure 11a that the corrosion rate of the cross section of Ex-AZ91-free alloy increased within 12 h of immersion, which illustrates the poor corrosion resistance of Ex-AZ91-free alloy. Figure 11b shows the corrosion rate of the longitudinal section of Ex-AZ91-Y alloy is faster than that of Ex-AZ91-free alloy. This reveals the corrosion resistance of the longitudinal section of Ex-AZ91-free alloy is better than that of the longitudinal section of Ex-AZ91-Y alloy.

### 3.7. Corrosion Morphology

**Figure 12:**
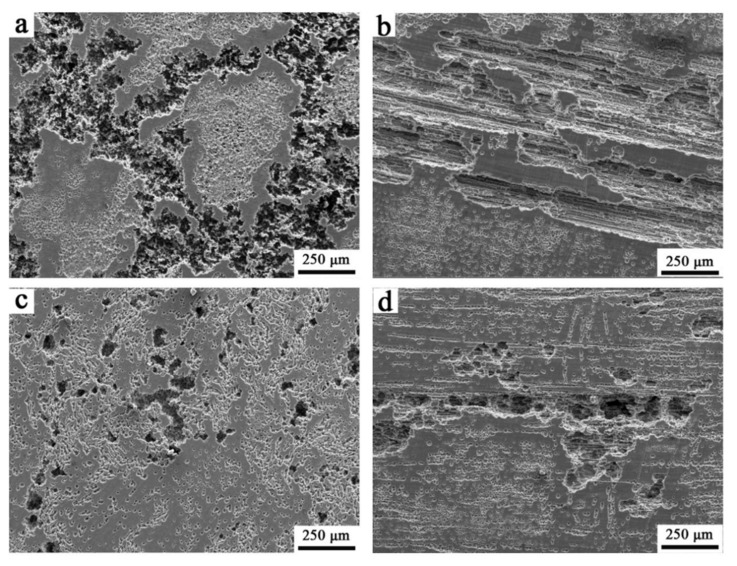
Corrosion morphologies of specimens after the immersion of 12 h, (**a**) transversal section and (**b**) longitudinal section of Ex-AZ91-free alloy; (**c**) transversal section and (**d**) longitudinal section of Ex-AZ91-Y alloy.

The corrosion morphologies of the transversal and longitudinal sections of extruded Mg alloy gained via SEM are demonstrated in Figure 12. The transversal sections of two extruded Mg alloys are subjected to different degrees of corrosion (Figure 12a,c). The corrosion of Ex-AZ91-free alloy is the most serious among them. Corrosion pits exist on the surface of the transversal sections of Ex-AZ91-Y alloy, but less than that of Ex-AZ91-free alloy. From the corrosion morphology of the longitudinal section (Figure 12b,d), it can be seen that the corrosion pits are distributed in streamline along the extrusion direction and the large corrosion occurred in the second phase enrichment region along the extrusion direction.

## 4. Discussions

It is generally acknowledged that the cathodic reduction reaction of Mg alloy mostly takes place in the second phase [17,37]. Hence, the cathodic reduction rate can be denoted in Equation (2) as follows:

*I*_*c*_ = *θ×i*_*c*_ (2)

where *I_c_* is cathodic reduction rate, and *θ* and *i_c_* are volume fraction and the current density of cathodic reduction process on the second phase of Mg alloy, respectively. It can be seen from Equation (2) that there are two factors affecting cathodic reduction rate. The volume fraction of the second phase of Ex-AZ91-Y alloy is significantly inferior to that of β phase in Ex-AZ91-free alloy (Table 2). The lower the cathodic Tafel slopes, the higher the cathodic activity [17]. Higher cathodic activity means higher current density of cathodic hydrogen evolution. The increased cathodic Tafel slopes and reduced volume fraction of the second phase of the transversal section indicate the decreased cathodic corrosion rate. Although the volume fraction of the longitudinal section in Ex-AZ91-Y alloy was lower than that of Ex-AZ91-free alloy, the cathodic current density of the second phase had a greater impact on the cathode reaction rate. Therefore, this provides a reasonable explanation for the phenomena that the corrosion resistance of the transversal section is different from that of the longitudinal section in Ex-AZ91-Y alloy.

The main reason for AZ91 Mg alloy corrosion is microgalvanic corrosion between the α-Mg and β phase under the condition of self-corrosion. Hence, the composition, size, quantity, morphology, and distribution of the α-Mg and β phase have a distinct influence on the corrosion behavior of Mg alloy. Song and Atrens [37] considered that the microgalvanic corrosion of Mg alloy depends on two factors: (1) the volume fraction and distribution of the second phase. The second phase as a barrier layer can hinder the corrosion of Mg alloy when it has a high volume fraction with distribution continuous in Mg alloy. On the other hand, the presence of the second phase can promote the microgalvanic corrosion of Mg alloy. (2) The cathodic reduction rate of the second phase. A higher cathodic reduction rate indicates the accelerated microgalvanic corrosion of Mg alloy.

According to Song et al.’s report [37], it is argued that the addition of Y has two different influences on micro galvanic corrosion of Ex-AZ91 alloy: On the one hand, Al, Mn, and Y can form the Al-Mn-Y phase after adding Y into Ex-AZ91 alloy. The formed Al-Mn-RE phase disperses in the Mg matrix and acts as a galvanic corrosion phase, which indicates the microgalvanic corrosion process is accelerated. On the other hand, the cathodic reaction rate primarily occurs in the second phase (Al-Mn-RE phase).

The above discussion shows for extruded AZ91-Y Mg alloy, the Al-Mn-RE phase can inhibit the cathodic reaction rate, which inhibits microgalvanic corrosion (a good effect). In addition, the dispersed distribution of the Al-Mn-RE phase can also accelerate microgalvanic corrosion (a harmful effect). This is in agreement with other literature reports [21]. The two factors compete with each other. When a good factor prevailed, the corrosion was inhibited, and vice versa. Connecting with the results of the polarization curve, EIS, and weight loss measurement, this discussion explains why the corrosion resistance of the transversal section is better than that of the longitudinal section in extruded AZ91-Y Mg alloy.

**Figure 13:**
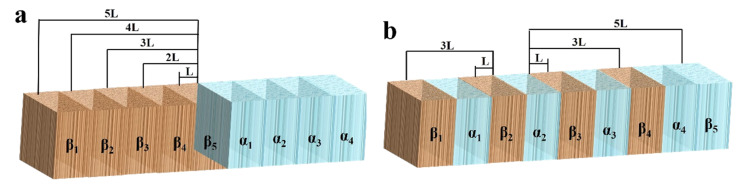
Diagrams of the model depicting the influence of the refined second phase on the microgalvanic corrosion of (**a**) Ex-AZ91-free alloy and (**b**) Ex-AZ91-Y alloy.

Now, many reports have focused on the effect of dispersion of the second phase on the corrosion of Mg alloy, but only a few have considered the influence of the refined second phase. According to Song’s research [38], the microgalvanic corrosion issue is simplified to a one-dimensional math problem and the theoretical expression of the galvanic potential and the current density of one-dimensional systems are inferred. Because of the complexity in geometrical shape of the second phase and α phase, it is actually quite hard to evaluate the galvanic current density and its distribution. Nevertheless, the refined second phase and α phase are divided into a nub and each piece is regarded as an individual [39]. According to this thought, a new simplified model (Figure 13) is proposed. The hypotheses about this model are proposed [39]:

1. The results of TEM manifest the size of the second phase to be about 0.5 μm in the Ex-AZ91-free alloy and about 100 nm in the Ex-AZ91-Y alloy. The size of the second phase of Ex-AZ91-free alloy is five times that of Ex-AZ91-Y alloy. Hence, the second phase of Ex-AZ91-Y alloy is considered as a signal piece and the second phase of Ex-AZ91-free alloy is assumed as five pieces.
2. It is supposed that the corrosion potential of the α and second phase, the polarization resistivity of the α phase in Ex-AZ91-free alloy and Ex-AZ91-Y alloy are same, and the *ΔE* is equal to the difference value between the second phase and α phase.
3. L is named as the distance between the piece of the second phase(β_1_) and α phase. Therefore, 2L, 3L, 4L, and 5L are the distances between the α phase and the piece of the second phase (β_2_, β_3_, β_4_, and β_5_), respectively.

On the basis of the model (Figure 13), if *I*_1_, *I*_2_, *I*_3_ …… *I*_7_, *I*_8_ are defined as the average galvanic current densities between the α phase and the second phase with the distances of *L*, 2*L*, 3*L* …… 7*L*, 8*L*, respectively, the galvanic current densities between the α phase and the second phase in Ex-AZ91-free and Ex-AZ91-Y alloys can be denoted to [39]:

(3) $$I_{1}\;=\;\frac{\Delta E\cdot [Cosh\left(\frac{L}{x}\right)-1]}{\rho_{s}Cosh\left(\frac{L}{x}\right)}$$

(4) $$I_{2}\;=\;\frac{\Delta E\cdot [Cosh\left(\frac{L}{x}\right)-1]}{\rho_{s}Cosh\left(\frac{2L}{x}\right)}$$

(5) $$I_{3}\;=\;\frac{\Delta E\cdot \left[Cosh\left(\frac{L}{x}\right)-1\right]}{\rho_{s}Cosh\left(\frac{3L}{x}\right)}$$

(6) $$I_{4}\;=\;\frac{\Delta E\cdot \left[Cosh\left(\frac{L}{x}\right)-1\right]}{\rho_{s}Cosh\frac{4L}{x}}$$

(7) $$I_{6}\;=\;\frac{\Delta E\cdot \left[Cosh\left(\frac{L}{x}\right)-1\right]}{\rho_{s}Cosh\frac{6L}{x}}$$

(8) $$I_{8}\;=\;\frac{\Delta E\cdot \left[Cosh\left(\frac{L}{x}\right)-1\right]}{\rho_{s}Cosh\frac{8L}{x}}$$

(9) $$I_{free}\;=\;I_{1}+2I_{2}+3I_{3}+4I_{4}+4I_{5}+3I_{6}+2I_{7}+I_{8}$$

(10) $$I_{Y}\;=\;8I_{1}+6I_{3}+4I_{5}+2I_{7}$$

*I_free_*, *I_Y_* are the micro galvanic corrosion of extruded AZ91-free Mg alloy and AZ91-Y Mg alloy, respectively.

(11) $$\begin{array}{cc} I_{Y}-I_{free} & =8I_{1}+6I_{3}+4I_{5}+2I_{7}-\left(I_{1}+2I_{2}+3I_{3}+4I_{4}+4I_{5}+3I_{6}+2I_{7}+I_{8}\right)\\ & =7I_{1}+3I_{3}-2I_{2}-4I_{4}-3I_{6}-I_{8}\\ & =7\frac{\Delta E\cdot [Cosh\left(\frac{L}{x}\right)-1]}{\rho_{s}Cosh\left(\frac{L}{x}\right)}+3\frac{\Delta E\cdot \left[Cosh\left(\frac{L}{x}\right)-1\right]}{\rho_{s}Cosh\left(\frac{3L}{x}\right)}-2\frac{\Delta E\cdot [Cosh\left(\frac{L}{x}\right)-1]}{\rho_{s}Cosh\left(\frac{2L}{x}\right)}\\ & -4\frac{\Delta E\cdot \left[Cosh\left(\frac{L}{x}\right)-1\right]}{\rho_{s}Cosh\frac{4L}{x}}-3\frac{\Delta E\cdot \left[Cosh\left(\frac{L}{x}\right)-1\right]}{\rho_{s}Cosh\frac{6L}{x}}-\frac{\Delta E\cdot \left[Cosh\left(\frac{L}{x}\right)-1\right]}{\rho_{s}Cosh\frac{8L}{x}} \end{array}$$

Because of the performance of Cosh(x) function, $I_{Y}-I_{free}>0$. On the basis of the model, it can be explained that the refined second phase can accelerate the microgalvanic corrosion and why an increase in the second volume fraction promotes the cathodic hydrogen reduction process.

From all the above discussions, it can be explained why the corrosion resistance of the transversal section in Ex-AZ91-Y alloy is stronger than that of Ex-AZ91-free alloy and why the corrosion resistance of the longitudinal section of Ex-AZ91-Y alloy decreases compared with the longitudinal section of Ex-AZ91-free alloy.

## 5. Conclusions

1. With the addition of Y, the β phase of Ex-AZ91 alloy is reduced and replaced by the Al-Mn-RE phase. Moreover, the volume fraction of the second phase in Ex-AZ91-Y alloy is lower than that of Ex-AZ91-free alloy.
2. The grains of Ex-AZ91 alloy were refined, the dislocation was not observed, and twin crystals reduced after adding Y.
3. The corrosion resistance of the transversal section of Ex-AZ91-Y alloy was improved compared with the transversal section of Ex-AZ91-free alloy and the corrosion resistance of the longitudinal section of Ex-AZ91-Y alloy was reduced compared with the longitudinal section of Ex-AZ91-free alloy. The improved corrosion resistance of the transversal section of Ex-AZ91-Y alloy can be attributed to the decreased volume fraction of the second phase and the decreased cathodic rate. The volume fraction of the longitudinal section of Ex-AZ91-Y alloy also reduced, but the accelerated cathodic corrosion rate of the longitudinal section had a more significant influence on the corrosion resistance, leading to decreased corrosion resistance of the longitudinal section.

## Figures and Tables

**Table 1:** Chemical composition of Ex-AZ91-free and Ex-AZ91-Y Mg alloy (%).

Element	Al	Zn	Mn	Fe	Si	Y	Mg
Ex-AZ91-Free	8.98	0.98	0.205	0.003	0.002	0	Bal
Ex-AZ91-Y	8.98	0.98	0.205	0.003	0.002	1.5	Bal

**Table 2:** The volume fraction of the second phase of Ex-AZ91 alloy with and without rare earth element Y.

Specimen	Ex-AZ91-Free Alloy (%)	Ex-AZ91-Y Alloy (%)
transverse	2.65	0.80
longitude	6.59	1.32

**Table 3:** The element contents of different intermetallic phase obtained by EDS.

Element	Point 1		Point 2	
	Wt%	At%	Wt%	At%
Mg K	62.77	65.45	0.86	1.46
Al K	36.43	34.23	39.65	60.34
Y L	0.25	0.07	21.94	10.14
Mn K	0.55	0.25	37.55	28.07
Matrix	correction	ZAF	correction	ZAF
